# Characteristics of effective entrepreneurship education post-COVID-19 in New Zealand primary and secondary schools: a Delphi study

**DOI:** 10.1007/s41959-022-00074-y

**Published:** 2022-07-26

**Authors:** Bethany Hardie, Kerry Lee, Camilla Highfield

**Affiliations:** The Faculty of Education and Social Work, The University of Auckland, Auckland, New Zealand

**Keywords:** Entrepreneurship, Enterprise, Innovation, Delphi, Education

## Abstract

This study was designed to investigate the perceptions of experts regarding the characteristics of effective entrepreneurship education in New Zealand primary and secondary schools. The aim of the study was to inform future policies, curriculum review and decision-making regarding entrepreneurial projects that were effective in the mainstream compulsory schooling sector. Using snowball sampling, 28 local entrepreneurship experts were recruited to participate in a Delphi Study. Through successive rounds, these participants established consensus on current and relevant characteristics of an effective entrepreneurship education primary and secondary school. The collective consensus determined seven characteristics for effective entrepreneurship education, centred around student learning approaches. Findings support curriculum planning focussed on creating authentic, action orientated projects or problem solving, strategies to foster entrepreneurship knowledge, skills and capabilities, seeking mentors or role models from the community, and the incorporation of financial literacy and business activities. These findings provide a basis for the successful development of New Zealand’s curricula for entrepreneurship education and enhanced entrepreneurship projects.

## Introduction

In this study, entrepreneurship education is defined as providing opportunities for students to develop an entrepreneurial mindset (Daniel, 2016) in order to learn skills to embrace, assess, and navigate new opportunities (Lackéus, 2015) in a dynamic and changing environment (Ratten & Usmanij, 2021). The popularity of entrepreneurship education first arose in the early 1980s in New Zealand as a way to grow the economy and the employment prospects of young people (Kirkley, 2017). In 2022, entrepreneurship education offers a classroom pedagogy for preparing students to tackle everyday complex and contemporary issues such as those associated with the restrictions imposed by the COVID-19 pandemic. Educational leaders need to consider the teaching environment required to prepare themselves and their students for new challenges presented by pandemic adjustments and rapid technological advancements (Mesquita & Vieira, 2020). Entrepreneurship education provides an opportunity for students’ capabilities to be fostered to be adaptable, resilient, innovative, resourceful and persevering (Lackéus, 2015) not only to cope with their disrupted schooling but to tackle the environmental, sociological, political and economic challenges they will face in their futures. An entrepreneurial education encourages and prepares students to find creative and innovative solutions to improve environmental sustainability (Rieckmann, 2020), transform social issues (Tapsell & Woods, 2010), and create successful small and medium businesses that can increase employment opportunities (Nseobot et al., 2020).

## Literature review

Research into how teachers and leaders have effectively implemented entrepreneurship education provides an understanding and theoretical framework of the experiences and pedagogical decisions that have enabled success. Establishing how the international literature describes effective entrepreneurship education in schools through a literature review, accumulated a vast array of definitions and characteristics that may not be generalisable to different contexts or take account of COVID-19 pandemic related factors (Hardie et al., 2022a). Entrepreneurship education has been applied within the school curriculum, either with a broad approach to instil general entrepreneurial capabilities in students (Lackéus, 2015) or with a narrow approach to study business start-ups (Huber et al., 2014). Students may learn ‘*about’* entrepreneurship by listening and reading, *‘for’* entrepreneurship by training in skills, or *‘through’* entrepreneurship experiences (Caird, 1990; Hannon, 2005). In addition to these well-known discrepancies in defining entrepreneurship education, the authors conducted a review of 17 studies that profiled the characteristics of entrepreneurship education and found that a wide variation in the number of characteristics used to define entrepreneurship education exists, ranging from 5 to 24 items per study (Hardie et al., 2022a). In order to understand the current knowledge of experts and determine an up to date description of the characteristics of effective entrepreneurship education in New Zealand schools, a Delphi method was applied.

### Expert evaluation of effective entrepreneurship education

We argue that perspectives from a wide range of stakeholders can help ensure ideas about proposed student learning objectives for entrepreneurship education are current and promote future success. In the past 2 years, current knowledge about how we deliver education and perceptions about how we encourage a buoyant and sustainable economy globally (**OECD, 2019, 2020), and in New Zealand has shifted (Robertson, 2020), compared to pre pandemic times. Public education now includes requirements for social distancing and more online learning and assessment (Zhou et al., 2020), while in the economic world entrepreneurship now traverses new challenges such as struggling supply chains, extended online shopping, changing vaccination requirements (Baker et al., 2020) and responsible sourcing that reduces our carbon footprint. It is therefore logical that utilising community networks to gain input into the most up to date knowledge for curriculum development in school-based entrepreneurial education is likely to result in relevant and sustainable ideas that will engage students interest and prepare them for their ongoing unpredictable futures (Crayford et al., 2012).

In the current study, a review of the literature found entrepreneurship experts are rarely consulted to define entrepreneurship education in research. Just two of 35 studies on entrepreneurship education consulted entrepreneurs within a school community as stakeholders to collaboratively define entrepreneurship education within a research project (Table 1).

**Table 1:** Methods used to define entrepreneurship education in 35 studies

Studies	Methods used to define entrepreneurship education (EE)				Number of studies with similar methods (total *n* = 35)
	Literature review	Local curriculum	Pre-established model of EE	Collaboratively defined EE in study	
Lackéus (2020), Hocenski et al. (2019), Abo-Shabana et al. (2018), Hanson (2018), Aladağ (2017), Kirkley (2017), Barba-Sánchez et al. (2016), Elo (2016), Hassi (2016), Lee et al. (2015), Testa and Frascheri (2015), Garnett (2013), Korhonen et al. (2012)	X				13
Fejes et al. (2019), Palmér and Johansson (2018), Jusoh (2012), Leffler (2012), Seikkula‐Leino (2011)	X	X			5
Pepin and St-Jean (2019), Pepin (2018), Birdthistle et al. (2016), Ruskovaara and Pihkala (2015), DeJaeghere (2014)	X		X		5
Floris and Pillitu (2019), Seikkula-Leino et al. (2019), Hämäläinen et al. (2018), Obschonka et al. (2017), Huber et al. (2017), Norberg (2017), Norberg (2016), Ishiguro (2015), Athayde (2012), Pihie and Bagheri (2011)			X		10
Ho et al. (2018), McLarty et al. (2010)	X			X	2

This international review found the most common methods used to define the characteristics of entrepreneurship education for further research were from literature or a pre-established model which draw on recent studies and models such as those from publications within the past 10 years.

### The rationale for a Delphi method

The Delphi method offers a relevant research method to define entrepreneurship education as it provides a process for reaching collective agreement between different entrepreneurship experts regarding classroom practice to meet the demands, needs and opportunities learners will face within society (Crayford et al., 2012). Experts in entrepreneurship education can include: leaders; teachers; students; parents; entrepreneurs and local businesses (Ruskovaara et al., 2016); local activists; non-government organisations; policy makers (Dahlstedt & Hertzberg, 2012); government officials related to education; researchers; primary, secondary and tertiary providers; entrepreneurship programmes; and social agencies (Kirkley, 2017).

In 1953, Norman Dalkey and Olaf Helmer first used the Delphi method to seek collective estimations on what the effect of an atomic bomb would be for the US military. It has since been used to gain the collective wisdom of experts for topics such as in education (Green, 2014), health education (Donohoe et al., 2012), optometry (Davey et al., 2017), architecture (Meijering et al., 2015), law (Guglyuvatyy & Stoianoff, 2015) and economics (Bernal et al., 2019). The Delphi method can define multifaceted, complex and context specific topics (Puig & Adams, 2018) such as entrepreneurship education by establishing consensus between entrepreneurship experts from a variety of backgrounds through iterative, anonymous rounds. Researchers can alter the logistics of the Delphi method to enable the collective contribution of participants from different geographic areas and time zones.

This paper outlines a Delphi study designed to establish the characteristics of effective entrepreneurship education in New Zealand schools in 2021 according to the current collective opinions of local entrepreneurship school leaders and business experts.

## Method

In this Delphi study, the authors employed purposive non-probability snowball sampling (Neuman, 2011) to approach 50 possible participants with relevant knowledge on entrepreneurship in New Zealand. Prior to the study ethical approval was sought by the authors and granted by the University of Auckland (UAHPEC22375). The inclusion criteria for selection of experts are shown in Table 2.

**Table 2:** Inclusion criteria for Delphi study entrepreneurship experts in New Zealand

*Included*
1. **New Zealand education or business-related academic staff** from universities in New Zealand who have published articles on entrepreneurship education or entrepreneurship. Identified by publication or referral
2. **New Zealand entrepreneurs** who have founded a company 10 + years old with significant turnover ($500,000NZ) and which employs 20 + people. Identified by referral, and the University of Auckland’s business school, or entrepreneurship club ‘Velocity’
3.**Government officials** related to entrepreneurship education from the Ministry of Education. Identified through Ministry of Education programmes and the Enterprise for Education (E4E) webpage
4.**Leaders of Entrepreneurship Education support programmes** for schools or universities in New Zealand. Identified through Enterprise for Education (E4E) webpage, Ministry of Education programmes, Young Enterprise, or the University of Auckland’s entrepreneurship club ‘Velocity’
5. **School principals** with interest in entrepreneurship. Referred by academics, entrepreneurs and government officials
*Excluded*
1. Experts who do not live in New Zealand
2. New Zealand education or business-related academic staff from universities in New Zealand who have not published articles on entrepreneurship education or entrepreneurship
3. New Zealand entrepreneurs who have not founded a company 10 + years old or without significant turnover ($500,000NZ) or who employ less than 20 people or not identified through contacts, the University of Auckland’s business school, or entrepreneurship club ‘Velocity’
4. Leaders for entrepreneurship education support programmes for schools or universities in New Zealand not identified through Enterprise for Education (E4E) webpage, Ministry of Education programmes, Young Enterprise, or University of Auckland’s entrepreneurship club ‘Velocity’
5. School principals not referred by academics, entrepreneurs and government officials
6. Members of the research team

Recruitment of participants relied on recommendations from contacts of potential participants and those who forwarded the email invitation to others with suitable expertise to contribute to the study. Potential participants were approached until identifiable experts were exhausted within a 2 week period. A teacher category was also included in the survey as it was predicted that referrals would lead to teachers being approached by contacts who knew them to be experts in entrepreneurship.

### Delphi study sample size

Delphi studies often experience a high drop off in participant numbers on each successive round of data collection. In a review of 19 studies utilising a Delphi methodology, a third (*n* = 7) experienced a marked drop in participation between rounds. To accommodate this drop off, Irdayanti et al. (2015) and McIntyre‐Hite (2016) recommend that 10 participants is the minimum sample size to ensure a standard of validity for a Delphi study. For this reason, a total of 28 experts in entrepreneurship (16 male and 12 female) participated in Round One and 20 experts in Round Two to evaluate the importance of specific indictors of an entrepreneurship education school (Table 3).

**Table 3:** The number of participants according to entrepreneurship background

Expert’s entrepreneurship background	Approached*	Round 1	Round 2
Academic researcher	11	3	1
Government official	14	1	1
Experienced entrepreneur	12	10	9
Leader from an entrepreneurship-related support programme	17	7	4
School principal	24	4	3
Teacher	3	3	2
Total	81	28	20

*Note*.*Not all referrals were disclosed by contacts to the researcher to protect people’s privacy

A total of 81 potential participants were approached through the snowball sampling recruitment process to recruit 28 participants.

Relevant academic researchers, government officials and entrepreneurs were harder to identify and recruit than other groups. Recruitment was higher for business entrepreneurs which solely relied on referrals rather than academic researchers and government officials who were recruited through website contact details. This is consistent with McIntyre‐Hite (2016) who explained that snowball sampling can improve recruitment and retention, as well as the quality of experts for a Delphi. Entrepreneurs in the current study were approached by a contact known to them, and all had experience in leading businesses with a turnover of between 30 million and 11 billion. Leaders from an entrepreneurship-related support programme were recruited through their websites and not by a snowball sampling referral by a known contact. One of these leaders from an entrepreneurship-related support programme referred five people for participation in the study whereby two principals and two teachers participated which demonstrated interest in the study. Although principals were the easiest to identify, they were also a group that was challenging to recruit for the study.

Most participants (*n* = 24, 86%) were older than 45 years of age, with 6 (21%) being over the age of 65 (Table 4).

**Table 4:** Age range of 28 experts in round one of a Delphi survey into entrepreneurship education

Age range	25–34	35–44	45–54	55–64	65–74
Frequency number (% out of 28)	2(7%)	2(7%)	11(39%)	7(25%)	6 (21%)

The age of most of the participants may be reflective of the time and experience required to reach the skill level required for the responsibility of leading schools and organisations.

### Delphi study rounds

A list of items for participants to rate was based on the findings of the literature review of 17 studies that had provided multiple characteristics of entrepreneurship education in primary and secondary schools (Hardie et al., 2022a). In a review of Delphi studies on education and entrepreneurship education, 15 of the 19 studies also provided a list of items for participants to rate. In the current study, the mechanism for collecting this information in Round One and Two was a 3–5 min survey (as stated in Table 5).

**Table 5:** The procedure for a delphi study on effective entrepreneurship education

Round	Method	Analysis
One	3–5-min survey (invited by email) which asked participants to: rate 25 characteristics of entrepreneurship education in schools on a 5-point Likert scale of importance, add extra characteristics, and list schools who exemplify the characteristics	The 25 characteristics were tallied according to the frequency of importance and ranked. Extra characteristics stated in the survey and explained by two participants who emailed feedback were summarised using thematic analysis into new items and listed below ranked items. List of effective entrepreneurship education schools was collated from Round One
Two	Summary of results of Round One reviewed by participants and feedback sought, before they completed a 3–5-min survey. In the second survey, participants ticked the ranked characteristics they believed were *most* important for the researcher to look for when identifying an effective entrepreneurship education school in New Zealand and were invited to list additional schools which fitted the characteristics	The frequency of ticks for each characteristic was tallied. The list of effective entrepreneurship education schools was collated from Round Two and added to the list from Round One

#### Delphi study analysis

In addition to identifying key features of effective entrepreneurship education experts determined were important, data analysis was undertaken to investigate whether variation in responses correlated with gender, age or experience; however, these were not found to be significant and therefore were not included in the findings.

## Results

### Delphi round one survey results

The sample size for this Delphi study was not large enough to allow for inferential statistics; therefore, descriptive statistics were used for analysis. The items were ranked according to the mean score of the experts' responses showing their perceptions about the importance of the characteristics as indicators of an effective entrepreneurship education in primary and secondary schools (Table 6). The importance Likert scales were converted to numerical values with not at all important to extremely important ranked 1–5, whereby a high mean value indicated higher regard of importance from experts towards the characteristics. New ideas or concepts of effective entrepreneurship education were added by nine participants, and thematic analysis was conducted on these to produce 11 new items for consideration in Round Two.

**Table 6:** Indicators of effective entrepreneurship education according to 28 entrepreneurship experts

Indicators of an effective entrepreneurship education school in average rank order	Number of experts *(n-28)*					*M*	SD
	Extremely important (5)	Very important (4)	Moderately important (3)	Slightly important (2)	Not at all important (1)		
Students’ entrepreneurship knowledge, skills, and capabilities are fostered	20	8	0	0	0	4.71	0.73
Students learn in authentic (real-world) contexts	21	5	1	1	0	4.64	0.72
There is innovative leadership that encourages and supports entrepreneurship education	17	9	1	0	1	4.46	0.88
Students are provided role models or mentoring for entrepreneurship	16	9	3	0	0	4.46	0.69
There is a flexible curriculum is designed to cater for entrepreneurship education	12	14	2	0	0	4.35	0.62
Management of change in pedagogy and curriculum is supported by leadership	10	17	1	0	0	4.32	0.55
Students frequently learn through action or problem solving to support the development of entrepreneurship education aptitudes	10	16	2	0	0	4.29	0.60
Students make decisions about their entrepreneurship education learning	11	13	4	0	0	4.25	0.70
Leaders, teachers and the school community have a shared common vision for entrepreneurship education	10	15	2	1	0	4.21	0.74
Leaders provide collaborative support and mentoring of entrepreneurship education	12	12	3	0	1	4.21	0.92
Parent, business and community are engaged in the entrepreneurship education programme development, design and delivery	10	15	1	1	1	4.14	0.93
There is a clearly articulated school culture of entrepreneurship education	10	11	6	1	0	4.14	0.76
A cross-curricular approach to implementing entrepreneurship education is supported by leaders and teachers	9	13	6	0	0	4.11	0.74
Entrepreneurship specific learning activities are consistently delivered as part of the curriculum	7	17	3	1	0	4.07	0.72
Entrepreneurial behaviour by staff and students is evident and supported	8	14	6	0	0	4.07	0.72
An entrepreneurship course is offered	9	13	4	0	2	3.96	1.07
Teachers act as facilitators of entrepreneurship education learning^a^	7	13	6	1	0	3.96	0.81
Staff work collegially to support entrepreneurship education	5	18	4	0	1	3.93	0.81
Entrepreneurship education assessment is focused on learning^a^	5	16	4	2	0	3.89	0.80
Students carry out business activities^a^	7	11	8	0	1	3.85	0.95
Teachers have had professional development in entrepreneurship education^b^	5	15	4	1	1	3.85	0.92
Students are frequently required to work in teams in entrepreneurship education^a^	6	12	8	1	0	3.85	0.82
Leadership have had professional development in entrepreneurship education	7	13	5	1	2	3.79	1.1
Strategic documents for entrepreneurship education are developed, shared and discussed at all levels of the school	8	10	5	4	1	3.71	1.15
Entrepreneurship education assignments require value creation^a^	2	17	6	2	0	3.70	0.72
New items:							
There is a school-wide broader focus on value for innovation, creativity and being enterprising							
Innovation, creativity, and flare are fostered in preparation for a dynamic context and ever-changing real-world environment							
Practical skills are taught through students creating a business in a fail-safe school environment							
A progression framework is provided for the entrepreneurial knowledge, skills and mindset to teach at each level of the curriculum							
Effective teaching of numeracy and literacy is provided							
Learning includes financial literacy							
There are links to outside agencies and community supporters							
There is Young Enterprise Scheme involvement							
The curricula distinguishes between entrepreneurship and social entrepreneurship							
It is ensured that there are visible champions in the school							
It is ensured that projects are real-world skills and experiences							

*Note:* Not all 28 participants answered every question. Means were calculated according to reduced participation with the number of participants to answer indicated by: ^a^*n* = 27, ^b^ *n* = 26

In completing the questionnaire, the participants suggested eleven new indicators of an effective entrepreneurship education New Zealand primary and secondary schools. These are noted in Table 6 under New Items.

### Delphi round two survey results

In Round Two, the Round One indicators were listed in the ranked order according to the average rating of importance and those indicators identified as ‘new’ were added to the list. Four of the eleven new indicators of entrepreneurship education suggested by the experts were notably similar to pre-existing characteristics which indicated experts may categorise or articulate items differently to the researcher. New items suggested by participants for example included “They learn practical, real-world skills (participant 8)” and “Real life applications (participant 12)” which the researcher would categorise within the item *students learn in authentic contexts*. Another new item suggested by participant 7 was that there is “A school wide focus on innovation and enterprising” which was similar to the item *there is a clearly articulated school culture of entrepreneurship education*. To ensure experts identified with the way items were articulated, these new items were not consolidated into pre-existing items and were listed in Round Two.

In Round Two, entrepreneurship experts were asked to tick the indicators they believed were most important for identifying an effective entrepreneurship education school. A total of 20 out of 28 experts in entrepreneurship from Round One participated in Round Two.

Characteristics that directly related to the quality and authenticity of student learning were seen as the most critical indicators of an effective entrepreneurship education primary or secondary school as shown in Table 7.

**Table 7:** Collective agreement of 20 experts on the critical indicators of an effective entrepreneurship education school (round two)

Indicator (frequency)	Participants in afgreement (%)
Students learn in authentic (real-world) contexts (16)	80
Student's entrepreneurship knowledge, skills, and capabilities are fostered (15)	75
Students frequently learn through action or problem solving to support the development of entrepreneurship education aptitudes (14)	70
Students are provided role models or mentoring for entrepreneurship (13)	65
It is ensured that projects are real-world skills and experiences (12)	60
Learning includes financial literacy (11)	55
Students carry out business activities (11)	55

Seven of the Round Two indicators listed in Table 7, had at least 55% agreement between experts that they were critical indicators which distinguished them from the remaining 35 characteristics which had fewer than 40% agreement.

Most experts agreed that *students learn in authentic (real-world) contexts* (*n* = 16/20, 80%) and *student's entrepreneurship knowledge, skills, and capabilities are fostered* (*n* = 15/20, 75%) and are critical indicators of an effective entrepreneurship education primary or secondary school (as shown in Table 7). As mentioned by two experts in the current study, entrepreneurship is dynamic, and the characteristics of authentic contexts and real-world skills in entrepreneurship education will depend on the schools community.

Some items remained important between experts in Round Two, these included *students frequently learn through action or problem solving to support the development of entrepreneurship education aptitudes* (*n* = 14/20, 70%, 3rd), *students are provided role models or mentoring for entrepreneurship* (*n* = 13/20, 65%, 4th), and *students carry out business activities (n* = 11, 55%, 6th)*.*

Two new items also attracted interest from over half of the experts as critical factors to an entrepreneurship education primary or secondary school was that *it is ensured that projects are real-world skills and experiences* (*n* = 12/20) and *learning includes financial literacy* (*n* = 11/20).

There was agreement between more than 75% (*n* = 15/20) of the experts that the remaining 21 characteristics were not critical indicators to identifying an effective entrepreneurship education primary or secondary school, suggesting much of what is articulated in the international literature was less relevant in the New Zealand schooling context.

## Discussion

The key characteristics for effective entrepreneurship education evident in this Delphi study provide New Zealand specific or ‘local’ expertise that suggests a strategic focus for future government and school level policies, to enhance a relevant curriculum that will advance student learning. Participants clearly stated that a priority focus for students is that they are engaged in learning in real-world contexts where their entrepreneurship knowledge, skills, and capabilities are developed through action or problem solving tasks. Participants clearly indicated their belief that students should have an opportunity to learn from role models or mentors and projects which involve real-world skills and experiences including financial literacy and business activities.

Five of the key characteristics for effective entrepreneurship education identified in this study support previous research which encourages the development of entrepreneurial capabilities by learning *through* entrepreneurship in order to foster students development of entrepreneurship knowledge, skills and capabilities. As Gibb and Price (2014) explain, effective teaching methods in entrepreneurship education seek to develop student’s entrepreneurial knowledge, skills and capabilities *through* experiential learning.

The New Zealand curriculum statement with its nine essential learning areas which is now 10 years old, supports school teaching programmes including a general focus on developing an enterprising mindset (Ministry of Education, 2011). The results of the study reported in this paper provide clear evidence that there is support for entrepreneurship education being strengthened through an integrated and authentic curriculum approach in primary and secondary schools, whereby, an integrated curriculum reflects the connections between subjects such as numeracy, literacy, science, and arts to develop student’s ability to consider multiple areas of knowledge in order to solve problems, create innovations and recognise opportunities (Fogarty, 1991) and relate learning to diverse student interests (Fitzpatrick et al., 2018). The results of this study support notions of teachers working together across subject areas to conceptualise these characteristics within units of work and in contexts for learning that incorporate real-world experiences. These would include action or problem solving tasks or projects, connecting to local role models or mentors, and include financial literacy and business activities. Planning could incorporate primary and secondary students connecting with the community and local businesses to meet mentors who can provide authentic contexts for learning and enhance the offerings within the school. These types of pedagogies support the recommendations of Fletcher et al. (2020), who argue that the provision of innovative learning environments and technology can further enhance a cross-curricular approach where by teachers can collaborate on planning, students can collaborate on work, and access to the experts is possible through conference calls.

A cross-curricular approach to education enables not only the development of student’s entrepreneurship competencies (Lackéus, 2015) but also their sustainable entrepreneurship knowledge to recognise opportunities with consideration for preservation of the environment, to address climate change and reduce pollution which requires knowledge from a range of subject areas (Strachan, 2018). Higher education now utilises networks more often for entrepreneurship embedded across faculties such as law, social sciences, the arts, and engineering (Crayford et al., 2012) with such initiatives also incorporating sustainable entrepreneurship (Rieckmann, 2020). Education has a responsibility to develop entrepreneurial citizens who recognise how decisions and opportunities may impact on the environment and cater for the growing preference of consumers for products and services responsibly sourced (Rieckmann, 2020; Strachan, 2018).

Leaders and teachers in primary and secondary schools need professional support to utilise networks to implement more authentic learning experiences and develop understanding in how to foster students’ entrepreneurship knowledge, skills and capabilities (Hardie et al., 2022b). In a previous study by the current authors, it was found resource allocation and professional development with the support of the whole school, school leaders or government funding and external networks better enabled entrepreneurship education (Hardie et al., 2022c). Without support, some primary and secondary teachers do not implement entrepreneurship education (Järvi, 2012; Neto et al., 2017; Winarno, 2016), use resources provided (Ruskovaara et al., 2015, 2016), or lack knowledge of effective pedagogy (Kamovich & Foss, 2017; Testa & Frascheri, 2015). Professional development has been found to improve teachers skill and frequency in implementing entrepreneurship education teaching methods (Ruskovaara & Pihkala, 2013, 2015), collaboration with peers and use of external networks (Ruskovaara et al., 2015). Classroom teacher knowledge may be strengthened by networking with local entrepreneurs with experience that will enable and inspire students to explore their creative ideas.

Local approaches to entrepreneurship need to take into account economic conditions and government COVID-19 response plans (OECD, 2021). A Delphi study by Anderson (2010) describes a benefit of a Delphi is that expert consensus can be establish in a short time frame and this was also found in the current study. The rapid changes of society to mitigate the spread of COVID-19 highlight how quickly literature, established curriculum and pre-established models of entrepreneurship that refer to a future with abundant opportunities have become outdated (Lee, 2021). Although the current study was carried out in the context of New Zealand, a Delphi would work well as a methodology to suit the uniqueness of communities across the globe because its relevance is strengthened by localised participants. As articulated by Sprott and Msengi (2020) in Alaska who carried out Delphi into developing a framework for multicultural education, a Delphi is a useful methodology for uncovering and giving voice to diverse viewpoints even within a single primary or secondary school.

The pandemic has also brought rapid changes in the economy and environmental viability of businesses and demonstrated the importance of developing students entrepreneurial tenacity to ‘pivot’ and adopt flexible ways of working throughout their life time. Therefore, by consulting entrepreneurship experts, primary and secondary school leaders and teachers can ensure that education is connected to the knowledge, skills and capabilities currently required of students and those for the projected future.

Equally, education itself has been challenged to pivot and respond following community isolation caused by COVID-19-related school closures. Entrepreneurship education offers the opportunity to reconnect (Flack et al., 2020) and reengage the primary and secondary school community with increased collaboration (Kirkley, 2017; Lackéus, 2020). Interviews with 18 New Zealand primary and secondary school leaders about the challenges of school closures found leaders increased their focus on well-being, effective communication, leading more collaboratively and enhanced opportunity recognition (Thornton, 2021). A survey of Australian (*n* = 2373) and New Zealand (*n* = 1183) teachers revealed that when 80% of schools experienced COVID-19 school closures, student–teacher and teacher–teacher relationships were crucial to supporting effective education and social wellbeing (Flack et al., 2020). Aspects of an entrepreneurship curriculum such as starting a small business or solving a community problem will often provide an opportunity for primary and secondary school teachers to better engage with students and their families within vulnerable student populations.

Where primary and secondary schools have once alienated the students family and home life, there has been a growing shift towards teachers and leaders incorporating authentic learning experiences from the home in order to recognise the rich learning experiences students encounter in their family and communities (Molina, 2013). More recently this shift has been accelerated by COVID-19 school closures, as Ng and Renshaw (2020) explain, although primary and secondary schools have been less able to connect with students during home learning, worthwhile learning experiences have happened within the cultures and traditions of families, transforming pedagogy to incorporate more connection between home and school. Brown (2020) suggests primary and secondary schools evolve to become community epi-centres where teachers, students and their families connect, where wellbeing is paramount, and where facilities offer spaces to meet and celebrate creative endeavours such as in art, music, sport, and technology.

The findings of this study are relevant to primary and secondary school leaders and teachers because the research clearly reveals that when entrepreneurial learning of students’ is made visible to the community through promotion, it is shown as being valued and recognised and this attracts support of potential community stakeholders. A study by Ruskovaara and Pihkala (2015) found that primary and secondary teachers who trained in entrepreneurship education used external stakeholders significantly more than peers. Networking with local experts was also found to be an effective approach to entrepreneurship education in a study by Neto et al. (2018). In a New Zealand or international context, showcasing how students are learning through entrepreneurship education on open days, the school website or publications may attract business partnerships, future employers, community and environmental groups to share and collaborate on ideas and provide students with authentic contexts for learning.

## Conclusion and contribution to knowledge

Public education has faced unforeseen challenges that have required a rapid response from governments and school leaders to adapt access to learning opportunities due to the COVID-19 pandemic restrictions given an exacerbated change to the local and global economic environment (Tarabini, 2021). This study reveals evidence that there is considerable agreement between New Zealand-based entrepreneurial experts in determining identifiable characteristics of an effective entrepreneurship education in primary and secondary schools with a focus on curriculum development. The results of this study offer a framework for developing achievement objectives and student learning outcomes for teachers and leaders to implement and strengthen what is currently a generalised approach towards entrepreneurship in the New Zealand curriculum. The multifaceted subject knowledge required for learning through entrepreneurship along with accommodating diverse student interests requires an integrated approach to the implementation of these characteristics through leaders and teachers utilising community networking to collaborate with entrepreneurs in their local community. These authors recommend the development of an integrated curriculum combining the social and physical sciences to enhance and foster environmental sustainability within entrepreneurship. In-depth research on effective entrepreneurship education in primary and secondary schools and the ways these programmes are implemented would contribute to understanding the underlying support that can enable student’s entrepreneurial learning and outcomes. Dissemination of the findings would further develop recommendations for a New Zealand focussed policy and curriculum that will enhance and transform the lives of our young people. However, given the advanced nature of the New Zealand education system and focus on localised curriculum, these developments will have international relevance in jurisdictions where social and environmental sustainability and entrepreneurship are at the forefront of economic viability.
